# Voices from the frontline: barriers and strategies to improve tuberculosis infection control in primary health care facilities in South Africa

**DOI:** 10.1186/s12913-018-3083-0

**Published:** 2018-04-10

**Authors:** Farirai Zinatsa, Michelle Engelbrecht, André Janse van Rensburg, Gladys Kigozi

**Affiliations:** 1 Centre for Development Support, University of the Free State, Nelson Mandela Road, Bloemfontein, 9300 South Africa; 2 Centre for Health Systems Research and Development, University of the Free State, Nelson Mandela Road, Bloemfontein, 9300 South Africa

**Keywords:** Tuberculosis, Infection control, Behaviour change, Healthcare workers, Primary healthcare facilities

## Abstract

**Background:**

Tuberculosis (TB) infection control at primary healthcare (PHC) level remains problematic, especially in South Africa. Improvements are significantly dependent on healthcare workers’ (HCWs) behaviours, underwriting an urgent need for behaviour change. This study sought to 1) identify factors influencing TB infection control behaviour at PHC level within a high TB burden district and 2) in a participatory manner elicit recommendations from HCWs for improved TB infection control.

**Method:**

A qualitative case study was employed. TB nurses and facility managers in the Mangaung Metropolitan District, South Africa, participated in five focus group and nominal group discussions. Data was thematically analysed.

**Results:**

Utilising the Information Motivation and Behaviour (IMB) Model, major barriers to TB infection control information included poor training and conflicting policy guidelines. Low levels of motivation were observed among participants, linked to feelings of powerlessness, negative attitudes of HCWs, poor district health support, and general health system challenges. With a few exceptions, most behaviours necessary to achieve TB risk-reduction, were generally regarded as easy to accomplish.

**Conclusions:**

Strategies for improved TB infection control included: training for comprehensive TB infection control for all HCWs; clarity on TB infection control policy guidelines; improved patient education and awareness of TB infection control measures; emphasis on the active role HCWs can play in infection control as change agents; improved social support; practical, hands-on training or role playing to improve behavioural skills; and the destigmatisation of TB/HIV among HCWs and patients.

## Background

Total eradication of the TB epidemic by 2030 is one of the key health targets of the post-2015 global TB strategy, underlined in the Sustainable Development Goal 3.3 [1, 2]. This target is reflected in the South African National Strategic Plan (NSP) for HIV, STIs and TB 2017–2022, which aims to reduce TB incidence by at least 30%, from 834/100,000 population in 2015 to less than 584/100000 by 2022 [3]. A step in this direction is addressing poor TB infection control in healthcare facilities. Research shows that poor infection control in healthcare facilities contributes to the high TB incidence [4–9]. Furthermore, active TB among healthcare workers (HCWs) in South Africa in 2015 was 1565 per 100,000 — more than double the notification rate in the general adult population [10]. Earlier research found that HCWs may even be up to three times more likely to acquire TB than the general population [8], while the risk for hospitalisation with multi-drug resistant (MDR) TB is approximately five to six times higher among HCWs than in the general population [11].

The comprehensive implementation of TB infection control measures, as proposed by The Centers for Disease Control and Prevention (CDC) and the World Health Organization (WHO), and adapted for and adopted into the South African Draft National Infection and Prevention Strategy for TB, MDR-TB and XDR-TB [12], is useful in mitigating the spread of nosocomial transmission [13, 14]. Appropriate TB infection control is dependent on early identification, isolation and rapid initiation of effective treatment of presumptive TB patients combined with good organisation in facilities to avoid overcrowding and ensure appropriate patient flow [15]. At the primary healthcare (PHC) level – the entry point for diagnosis, treatment and management of TB and MDRTB in South Africa —the implementation of TB infection control is largely dependent on nurses [16]. Several studies, however, point to poor TB infection control practices at PHC level [7, 16–21].

**Fig. 1:**
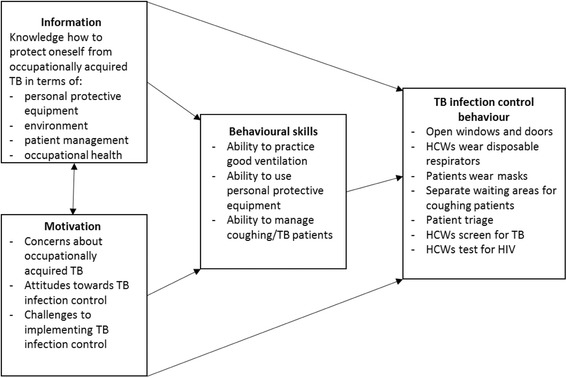
Adapted IMB model for TB infection control

Indications of inequities and fragmentation in terms of inter-provincial and regional health system performance in South Africa [22, 23] call for TB infection control interventions with a good level of contextual fit, that include the voices of local implementers and supporters [24–27]. The bottom line is; behaviour change at PHC level is necessary to address poor implementation of TB infection control. In this regard, research is required to identify behaviour change strategies to improve practices in PHC facilities. This study applies the Information Motivation and Behavioural Skills (IMB) Model [28] to identify factors that influence TB infection control practices and elicit recommendations to improve infection control in PHC facilities (see Fig. 1).

The IMB model is a social psychology model used to both understand and promote health-related behaviour. The model proposes that individual, information, motivation and behavioural skills are important factors to achieve risk-reduction and positive health outcomes - but more so when utilised in tandem [28]. The IMB model has been used to guide the development of health behaviour interventions based on empirical evidence in TB treatment adherence, diabetes [29] and HIV risk reduction [29–31]. However, it has not been used to specifically develop interventions for TB infection control. Close engagement with theoretical frameworks promote intervention fidelity and effectiveness, by assisting researchers to identify mechanisms that influence intervention success, increasing understanding of the contextual factors that may drive actual implementation [32, 33].

## Methods

### Design

Epistemologically the research was positioned in a qualitative paradigm. More specifically, it followed an interpretive approach. It also followed a critical approach in so far as the research aimed to address a pressing healthcare problem in a participatory manner, which allowed for inclusion of on-the-ground stakeholders and shared decision making. More specifically, a qualitative case study approach was undertaken.

### Setting

The research was undertaken in a high TB- and HIV-burdened metro, one of seven metropolitan areas, in South Africa. The study builds on the findings from a survey conducted in the metro during 2015, which investigated the status of TB infection control in 41 PHC facilities [20]. Based on our observations, at the time of the 2015 survey, there were 43 PHC facilities in the metro, 41 of which diagnosed TB in patients, and of these 39 provided monthly follow-up treatment and care for TB. The facilities were mainly staffed by professional nurses, with an average of 5.6 professional nurses per facility. The TB incidence, 686/100000 population, and HIV prevalence among 15–49 year-old women attending antenatal care, 30.4%, was higher in the metro than the country as a whole (593/100000 population and 29.7% respectively) [34, 35]. Furthermore, there are indications that TB infection control is sub-optimal in some public health facilities in the metro [20, 36, 37].

### The research team

The research team, conceptualised the study, developed the interview guide, conducted the focus group discussions, analysed the data and drafted the article. The team comprised of two senior researchers and one researcher, all of whom specialise in health systems research with a focus on the social aspects of TB - for more than a decade. In addition, the team also included a student completing her Master’s in Development Studies. The three researchers also undertook the 2015 TB infection control survey in the Metro and had a professional relationship with the PHC managers and TB nurses who participated in the study.

### Participant selection

In the current study, the 41 PHC facility managers and 41 TB nurses who participated in the 2015 TB infection control survey [20] were purposively selected to take part in focus group discussions to identify strategies to improve TB infection control. In total, 33 facility managers and 20 TB nurses agreed to participate in the focus group discussions. Eight facility managers were not available on the day of the focus group discussions and given substantial nursing shortages, it would have been unethical from the researchers to take any more nurses out of the system to participate in the research.

### Instrument development

The IMB Model provided a theoretical framework to guide the enquiry and was used for data collection as well as data analysis. The IMB Model was first developed by Fisher and Fisher [28] and was initially used to study risky behaviour related to HIV infection, but has since been expanded to include a range of different risk behaviours [29]. Information refers to basic medical knowledge about a specific condition, including disease development and management strategies. Motivation comprises personal attitudes towards adherence behaviour, perceived social support as well as perceived notions regarding the behaviour of those afflicted by this condition. Behaviours include specific behavioural strategies and skills required for adherence behaviour and self-regulating behaviours. It should be noted that all three components should directly pertain to the desired outcome, in this case, TB prevention through appropriate infection control [8].

Key texts [12, 16, 36, 38–40] as well as the IMB model informed the development of the focus group discussion schedule. The questions were designed to cover all levels of TB infection control: facility, administrative and environmental controls, as well as the use of personal protective equipment. The focus group discussion guide was divided into three sections, each aligned to the key factors outlined in the IMB model: information, motivation and behaviour. The aim of the discussion was to identify factors that impede and/or facilitate TB infection control in PHC facilities. More specifically, the questions relating to information focused on knowledge of: the different roles of district TB managers, facility managers and TB nurses in ensuring TB infection control in PHC facilities; how HCWs can protect themselves from TB infection in terms of using personal protective equipment, appropriate ventilation, triaging patients and screening for TB; and training on TB infection control. Questions on motivation to implement TB infection control focused on: concerns about occupationally acquired TB; knowledge of co-workers with TB; attitudes towards implementing TB infection control; provincial and district support for TB infection control; and TB stigma. The focus of the behaviour questions was on challenges with implementing good TB infection control practices, including keeping: windows and doors open in the PHC facility; wearing a disposable respirator while working with TB patients; triaging coughing patients.

In addition, questions were also developed to reach consensus on strategies to improve TB infection control in PHC facilities. These questions related to improving: the use of disposable respirators for HCWs and masks for patients; triage of coughing patients; and ventilation by keeping windows open. There were also questions on: the best way HCWs can protect themselves from TB infection; training for TB infection control; and incentives for good practices. These questions were applied using nominal group technique. This particular data gathering method is a structured small-group discussion with the principle aim of consensus building; responses to specific questions are elicited by a moderator, after which emerging ideas are prioritised [41].

### Data gathering

The five focus group discussions with PHC facility managers and TB nurses were conducted during September 2016 to identify factors that impede and/or facilitate TB infection control in PHC facilities. Each group had between 7 and 10 participants, and the discussions lasted approximately 90 min. The discussions were conducted in private rooms and refreshments were provided after the completion of the discussions. More specifically, one focus group was held with only TB nurses, three with facility managers, and one combined group of facility managers and TB nurses. Only one dedicated group of nurses were included – given substantial nursing shortages – it would have been unethical for the researchers to take more nurses out of the system. Regarding the variation, it was necessary to differentiate between nurses and managers, as well as having one mixed group, in order to account for possible differentiation across groups, and to contain in-group dynamics according to nursing hierarchies. For example, nurses may act differently in a group with managers than in a dedicated nursing group. It should also be kept in mind that facility managers provide nursing services, including TB care, at PHC facilities with the added responsibility of managing the facility.

Prior to commencing with the discussion, informed consent was obtained from all participants, who were also asked to complete a short questionnaire capturing demographic information. A moderator and note-taker facilitated each focus group discussion, which was also audio-recorded with participants’ permission. Data saturation was negotiated amongst the researchers, and data collection continued until saturation was reached.

Immediately following the focus group discussion, the nominal group technique [42–44] was used to reach consensus on strategies to improve infection control in PHC facilities. The moderator posed questions related to specific challenges in TB infection control, and asked participants to write down, on a piece of paper, the most important idea about how to solve each challenge. During this time, participants were asked not to discuss any ideas. The moderator collected all pieces of paper and pasted them on a board for everyone to see. An open discussion followed, where the ideas offered by individual participants were scrutinised and ranked until consensus was reached. This process was repeated until key recommendations were developed to strengthen TB infection control.

### Data analysis

Data from the focus group discussions were analysed using thematic analysis. In this way, summative, phenomenological meanings of text were identified that represent lived experiences, categorised according to a theoretical model (deductive approach), and constructed from repeated reading of the transcripts (inductive approach) [45]. Audio-recordings from the focus group discussions were transcribed verbatim and supplemented with notes taken during the discussions. The resulting data were manually analysed, guided by Braun and Clarck’s [46] stages of thematic analysis. Transcripts were read through several times to identify initial themes. Once the themes were identified, statements from the participants were sorted in line with the themes. The statements were then grouped into themes and sub-themes based on the IMB model. This process resulted in the identification of three deductive themes (information, motivation and behaviour), and two inductive themes (district and provincial support). The last stage of the analysis involved integrating the two inductive themes on the premise that these fitted into the motivation theme.

Inter-rater reliability was followed, in that multiple researchers negotiated among themselves the number and content of themes that emerged [47]. The researchers compared their thematic categorization in an iterative way, resulting in on-going processes of verification and corroboration, elevating the quality of the analysis process. The subjectivity of researchers was a key part of the analysis. In qualitative research, researchers must draw from their own subjectivities in order to objectively reflect on social phenomena, in this case, TB infection control knowledge and practices. More specifically, the researchers’ subjectivities of TB prevention interrogated the perspectives of participants towards forming a more objective illustration [48]. Notable quotes were used to illustrate participant responses across themes and sub-themes. Arguments and quotations from different participants added another level of transparency and trustworthiness to the data.

In the analysis, distinction were not specifically made between the facility managers and TB nurses, as facility managers also provide TB services with the added responsibility of being a manager. In instances where certain aspects of TB infection control is clearly the manager’s responsibility, this is highlighted as such.

The nominal group technique process did not aim to generate data for thematic analysis. The nominal group technique sessions which formed an adjunct to the focus group discussions mainly provided participants with an opportunity to brainstorm and agree on what measures would improve infection control practices in response to very specific questions on TB infection control.

### Ethics and authorisation

All participants in the study provided written informed consent, and all data was anonymised during analysis. As confidentiality cannot be guaranteed in group-based research, participants were asked to keep information discussed during the sessions confidential. Ethical approval for the study was obtained from the Faculty of Humanities, University of the Free State (Ethics reference number: UFS-HSD2016/0687). Approval for the study was also obtained from the Free State Department of Health.

## Results

The findings are aligned to the three themes of information, motivation and behaviour as well as to the hierarchy of TB infection control [12, 13]. The anonymised source of direct quotations is indicated by parentheses at the end of each quote. The following abbreviations are used when referring to the source transcripts; namely, BbO1/BbO2 – Botshabelo focus group discussions, BLM01 – Bloemfontein focus group discussion, TbN01/TBN02 – Thaba Nchu focus group discussions.

In total 33 facility managers and 20 TB nurses participate in the focus group discussions. There were 45 female and 8 male participants. The average age of participants was 51 years, and they had been working in the medical profession for an average of 24 years.

### Information

With regard to facility and administrative controls, training, policies and poor knowledge were found to have an influence on TB infection control implementation.

### Training

A number of the participants stated that they had not received any formal TB infection control training. It was noted that when TB infection control training was provided, it was mainly for facility managers, “Most of the time [training] it’s for the managers. For the lower category is very scarce. You get it after a long, long time… and you are the one who is working at TB control” (TbN01). In addition, when TB infection control training was offered for TB nurses, there were reportedly significantly long lapses between training and refresher training. Participants would like to receive TB infection control training in the form of off-site training workshops, followed by in-service training and mentoring. Routine refresher courses were also suggested. It was also highlighted that TB infection control was not only the responsibility of nurses, therefore training should be provided for all PHC workers including data capturers, cleaners as well as patients just as it was done for HIV, because “…TB is not for nurses, it’s for everybody and also the patients.” (TbN1).

### Policies

Various concerns were raised regarding TB infection control policies and guidelines at PHC level. For example, participants were confused by numerous policies, some of which were still in draft form. Furthermore, it was noted that there were conflicting guidelines in some policies: “You know what, the National Core Standard says this, the Ideal Clinic says this, so you as a middle man being an operational manager you end up being confused because the Ideal Clinic says you must not have a separate TB room, the TB falls under chronic patients. The National Core Standard says you must have a TB cubicle, so..?” (BLM1). One participant also expressed doubts about guidelines prescribed by the Ideal Clinic policy: “They say if someone is on treatment then he is no more infectious, so really we query that but we don't know” (BbO2). Other challenges included the perception that some policies were not applicable to PHC settings, “… when you read the policy you see that it doesn't affect people at the primary healthcare level…it's for hospital based - that's the problem” (BbO1).

A further challenge was that participants did not always understand the policies, “…because these policies are only imposed to us and [we are] not trained on them, sometimes when we read them for the subordinates sometimes, we don't understand it.” (BbO1). Reportedly, in-house TB infection control training, including policy training, was one of the main responsibilities of facility managers; however, most of managers reported feeling ill-equipped to undertake such training: “It's for us as managers to sit down with your staff, even if you don't understand it [the policy] yourself, you've gotta read with them [TB nurses] and then make them to understand” (TbN02). In this regard, one participant expressed the desire to be involved in policy decision making, “… even though we are not policy makers but we [facility managers and TB nurses] must... be involved during that policy making decision.” (BbO1).

### Incorrect information/lack of knowledge about personal protective equipment

Incorrect information and/or a lack of knowledge about personal protective equipment influenced the implementation of TB infection control guidelines. Many participants were unable to explain why HCWs used disposable (N95) respirators and why patients who coughed used masks - “I think the most important part, we spend most of the time in the clinic that’s why we use the N95, and the patient goes in and out that’s why he or she is supposed to use a surgical mask.” (TbN01). Some participants also lacked knowledge about the reuse and storage of N95 respirators. One TB nurse asked, “What is the good practice?....So the only time when you are supposed to discard it is when it doesn’t fit you nicely, it's loose and dirty. It's then that you can discard it” (TbN2). Some participants thought that the same disposable respirator could be used for several months while others felt that disposable respirators should only be used only once. One participant stated that she had been taught by the infection control nurse that she could wipe the outside of a disposable respirator with a clean cloth in order to make it fit for reuse. Furthermore, the majority of participants did not know what fit testing of a disposable respirator entailed: “I really thought that the fitting was just to press it here. I even learned [taught] the people when they asked me how do you fit it, I said you just take it and you press it here. I really thought that’s what they mean by fit” (BLM1).

### Factors motivating HCWs to implement good TB infection control

Several factors reportedly encouraged implementation of TB infection control measures and included: knowing a colleague who had fallen ill with TB, role-modelling by facility managers, mentorship, having a patient with multidrug-resistant (MDR) TB, National Core Standard inspections, and workmen’s compensation.

### Colleague with TB

Knowing a colleague with TB raised awareness of the risk for nosocomial TB transmission: “Maybe it has taught us the reality because now working in most cases we don't protect ourselves. We don't wear N95s as we are supposed to. Maybe it's a wakeup call to say we as HCWs we are not immune to TB. We can also get TB” (BbO1). It also encouraged improved infection control: “…after that nurse was infected with TB we had to move to the smaller room but at least it has two doors and then we decided that our patient[s] must wait outside in the line then it improved. No one got infected after that.” (BbO1). However, the motivational strength of knowing a colleague with TB was mitigated by concerns over health system related challenges, such as poor working conditions “… the working conditions still stays the same. Yes, it [the TB in the colleague] was actually induced by the working conditions and the structures of our facilities.” (BbO2). The motivational impact was also reduced by the belief the colleague could have been exposed to TB outside of the healthcare setting, “…but if the colleague has TB or any [other] infections, we don't have the guarantee that he or she got it from the workplace.” (BbO2).

### Managers as role-models

Facility managers saw themselves as an important source of social motivation by modelling appropriate infection control practices, such wearing of disposable respirators and encouraging compliance with TB infection control protocols, “We just have to encourage them [TB nurses] to use them [N95s] on daily basis not only when they serve MDR patients or when there is an inspection.” (BbO2). In addition, support and mentorship from district officials was regarded as being particularly important in motivating compliance with TB infection control guidelines. As one participant stated “…they must support the facilities to see whether are they doing the right thing… just to check are we following the correct measures.” (BLM1).

### MDR TB patients, inspections and workmen’s compensation

Some participants were more likely to comply with the use of personal protective equipment when they knew that a patient had MDR TB or when they knew that a National Core Standard inspection was imminent. Workmen’s compensation for falling ill with TB was also regarded as a motivator for TB screening - as indicated by one staff member: “…cause I make them aware that if you can contract the TB and you don't have the previous findings that you were negative you are not going to be compensated….. it [compensation] is a motivator for them [TB nurses] to take the sputum, do screening, but for HIV, it's confidential” (BbO1).

### Factors impeding HCWs to implement good TB infection control

Barriers to good TB infection control were categorised as personal, occupational and health systems related.

### Personal factors

Personal factors impeding HCWs from implementing good TB infection control practices included feelings of powerlessness and poor attitudes.

Feelings of powerlessness manifested in various ways. Facility managers’ statements suggested that they felt powerless to do anything to prevent TB infection within healthcare facilities. In what appeared to be a reference to their hierarchical position in the provincial Department of Health, one facility manager remarked, “We are concerned [about contracting TB] but there is nothing that we can do. Yes. It's beyond us. We are just managers down there. We are really, really low.” (BLM1). Some participants appeared to be in complete denial about the possibility of contracting TB: “What I mean is that you know…I don't know how to put it… we don't get it really. God protects us really. Really we don't get it. It is very rare.” (BbO1). While other participants, believed that most HCWs had already contracted TB/MDR-TB. Feelings of powerlessness also seemed to arise from being unable to prove that TB had in fact been contracted within the healthcare facility. One participant explained: “If you get TB at the work, you must prove to them that you did not have TB before or that you didn’t get it in the Hyperama. It's impossible to prove that you got TB from your environment, wherever! It's impossible really!!” (BLM1).

There appeared to be a general consensus among participants that most HCWs in PHC facilities had negative attitudes towards their working conditions, as reflected in one participant’s statement: “The people are very negative. This is such longstanding problems in the primary healthcare that even if you tell me it will change [by] December I will think it's a big joke!!” (BLM1). In addition, it was also noted that TB nurses were negligent; as one participant explained, “….but again they [TB nurses] are negligent. Others we’ll be seeing just wearing [an N95 respirator] on the forehead like a cap, just on the forehead. Negligent also.” (BbO1).

### Occupational factors

Some participants voiced the concern that even if TB infection control practices were well implemented, it did not guarantee that a HCW would not become sick with TB. There was a general perception that PHC facilities were neglected and received less support than hospital facilities. Poor occupational support at PHC level manifested in the lack of fit-testing of N95 respirators as well as the lack of routine screening of HCWs for TB and HIV. Furthermore, participants thought that they would be accused of negligence if they became infected with TB. These challenges resulted in reluctance to voice concerns; as noted by one participant: “…there is no support for us…sometimes even when you have pricked yourself with the needle, sometimes you don't do anything. You say God is there….” (BbO2).

There was the perception that even if TB screening led to a HCW being diagnosed with TB, not much would be done about it, “….because nobody cares, even if [I am] identified on diagnosis I must die, it doesn't matter” (BLM1). Some participants were of the opinion that screening was not worthwhile due to its low yield of TB positive patients/HCWs, “I think we would have picked them [TB suspects] up in any case because they were sick or had the symptoms of TB because we always knew what were the symptoms of TB were. So I don't know if that screening has really effect on anything but….… you screen all these people and the percentage of people that you get that are still positive is not so high” (BLM1).

### Health system factors

Health systems factors hampering good TB infection control included poor facility infrastructure coupled with poor maintenance, as well as a lack of material and human resources.

There was a general consensus among participants that infrastructure was one of the greatest impediments to good TB infection control implementation, as one participant stated, “… the current infrastructure in most of the facilities that's the biggest challenge.” (BLM1) Specific problems included: PHC facilities were too small, leading to congestion and overcrowding of waiting rooms; a lack of separate waiting rooms for TB patients and general patients, as shown in the following statement, “….it's only one waiting room and it is difficult for me to separate the TB patients and the other patients.” (BbO1). A few facilities had outside shelters to accommodate TB patients but this was the exception. It was also noted that there were windows that did not open as well as air-conditioning units and ultraviolet germicidal (UVG) lights which had not been serviced for a considerable length of time. Further health system challenges included the lack of materials/equipment (i.e. disposable respirators, surgical masks, tissues, hand soap, and cleaning materials) as well as the shortage of human resources for PHC.

### Behaviour

Many of the practices required for good TB infection control were regarded easy to implement by most participants. Tasks that participants found difficult included fit-testing and donning of disposable respirators as well as disclosure of one’s HIV status. It was also noted that certain TB infection control practices were associated with stigmatisation of patients.

It is a concern that fit-testing of disposable respirators was not done, as one participant explained: “We don’t do fit testing. We just order and it comes then everyone puts on…it has that silver thing that you press here then it fits. All the N95 have got that…” (BLM1). Participants also raised the issue that disposable respirators were uncomfortable to wear; they struggled to breathe and felt that they were suffocating. Patients, also found it difficult to understand what HCWs were saying when they wore disposable respirators. Surgical masks were reportedly much more comfortable to wear, even though they did not offer an adequate level of protection for HCWs.

The implementation of environmental controls was hampered by patients who closed the clinic doors and windows particularly during cold weather. Stigmatising infection control behaviours were also reported, for example, the separation of coughing patients, particularly when they were made to stand outside: “No, [the TB patients wait] outside. Outside the building, in the stoop and others feel neglected. It is discrimination. [They feel] very bad, very bad. No, they don’t like it.” (BbO1). Asking coughing/TB patients to wear a mask also created stigmatisation: “Even if you give them mask to use they feel discriminated. It’s TB. Now you want to show others that they have TB. So they will never use it.” (BbO1). The participants also expressed the view that patients were afraid to enter the TB room because of the association between TB and HIV: “...because they are afraid people saying that if you get in the TB room meaning you are HIV positive” (TbN2).

### Strategies to improve TB infection control in PHC facilities

The nominal group technique sessions provided participants with an opportunity to brainstorm and agree on what measures would improve TB infection control practices in response to very specific questions on infection control. There were some notable recommendations as to how TB infection control implementation could be improved (see Table 1). At the managerial level, it was recommended that district and provincial managers should ensure that PHC facilities had sufficient resources to implement TB infection control, and that policies should be clear. It was also noted that there should be an award (e.g. a trophy or certificate) for the TB nurses who best implemented infection control in their facilities. It should, however, be kept in mind that during the focus group discussions, one participant stated: “Incentives? Incentives for who? No, what incentives? We don’t get any incentives for anything. There are no incentives. For good practices? Hah, never!!” (BLM1).

**Table 1:** Recommendations for improving TB infection control

TB Infection control practice	How it can be improved
Managerial level	
What should be the District and Provincial Managers’ Role in improving TB infection control?	1. Ensure provision of material resources 2. Ensure policy clarity and implementation 3. Training/Mentoring and consultation to facilities 4. Provision of adequate human resources 5. Monitoring and Evaluation 6. Improve infrastructure and maintenance
What possible incentives can be introduced for the good practice of TB infection control?	1. Award for TB focal person 2. Prize-giving for facility e.g. trophy 3. Counselling and occupational health support
Administrative level	
How can we promote the use of tissues/masks for patients?	1. Health education/group information sessions or videos 2. Provision of masks (in waiting area)
How can we separate coughing from non-coughing patients?	1. Triage and fast-tracking
How can we promote TB and HIV screening for HCWs?	1. Education on importance of screening 2. Routine screening by the occupational health nurse at central sick bay with record keeping 3. Incentives 4. Counselling
What is the best format for TB infection control training?	For HCWs: 1. Workshop training followed by in-service/practical training at facility 2. Mentoring 3. Refresher training For patients 1. Facility videos to educate patients 2. Reading materials such as pamphlets for patients
Environmental level	
How can we ensure the windows are opened and kept open in waiting areas and consultation rooms?	1. Allocate open-window marshal/focal person 2. Utilise monitoring tool, i.e. window register 3. Patient education and engagement 4. Keep open window posters/stickers
Personal protective equipment	
How can we promote the use of disposable (N95) respirators among HCWs?	1. Provide disposable (N95) respirators and fit test 2. Training/education 3. Ensure compliance/Discipline (written warning on record) 4. Ensure policy is carried out

At the administrative level it was recommended that the best format for TB infection control training was formal workshops followed by in-service training and mentoring. It was noted that all HCWs at PHC facilities, not only managers and TB nurses, should receive training in TB infection control. In addition, HCWs should be educated on the importance of screening for TB and HIV. Patients also required education on the importance of using a tissue or mask to cover their cough. At the environmental level, it was noted that a queue marshal should be appointed to ensure that doors and windows remained open during the day and also to explain the importance of natural ventilation to patients. Finally, it was suggested that facilities be stocked with disposable (N95) respirators and that HCWs be taught how to fit test the respirator.

## Discussion

Poor implementation of TB infection control practices remains a challenge within PHC facilities. We used the IMB model to identify factors influencing TB infection control practices by HCWs in Mangaung Metro. The model suggests that individuals who are knowledgeable, sufficiently motivated, and who are in possession of the necessary behavioural skills, are most likely to maintain health-promoting behaviours with positive outcomes [28]. Comprehensive and sustained implementation of TB infection control protocols by HCWs is key to reducing the risk of nosocomial transmission of TB and this is best achieved through behaviour change. Our study is unique in that it employed a participatory approach in exploring recommendations for improved TB infection control practices by HCWs in PHC facilities. In doing so, it took the realities of HCWs into context and allowed for the voices of those involved in TB infection control at the grassroots level to be heard. As noted by Gilson [49], it is the frontline workers who “ultimately translate policy intentions into practice”, and this speaks to the importance of involving PHC nurses and managers in decision making. Despite this, there is a dearth of studies that explore nurses’ participation in broader health workforce policies [50]. Hence our study adds value as it allows PHC nurses and managers to provide their insights into strategies that could improve TB infection control.

Information-related barriers to good TB infection control, included poor training, incorrect knowledge and critical knowledge gaps – which was not unique to our study [20, 40, 51–53]. The finding that TB infection control training mainly targeted PHC facility managers and not TB nurses, is in stark contrast to guidelines from the National Department of Health [12] and other recommendations [54] which endorses training for all HCWs including lay HCWs. Despite being expected to train staff on TB infection control, PHC facility managers indicated being inadequately prepared to do so.

Conflicting guidelines, in particular the National Core Standards and the Ideal Clinic Policy, contributed to confusion and poor implementation of TB infection control measures. The National Core Standards are used as a benchmark for an “an expected level of performance” by which public health facilities can be assessed [55]; while the Ideal Clinic Policy guides transformation of all PHC facilities so that they comply with standards set out by the Office of Health Standards Compliance (OHSC) in order to prepare for the roll-out of National Health Insurance (NHI) [56]. The contradiction between Ideal Clinic and National Core Standard guidelines relates to the separation of patients, which is regarded as a key administrative measure to reduce the risk of nosocomial transmission within healthcare facilities [12].

Personal motivating factors centre on the beliefs and attitudes of HCWs which, together with social motivation [29], are key to implementation and adherence to infection control guidelines [33]. Nurses are at the forefront of TB infection control implementation within PHC facilities and therefore it is critical that they are adequately motivated. Contrary to other studies, which established appropriate levels of motivation among HCWs [39], we identified low levels of motivation. Feelings of powerlessness, negative attitudes of HCWs, poor district health support, and poor occupational health support, were identified as impediments to the implementation of TB infection control. Belief in the lack of care and concern for PHC as evidenced by “longstanding” health system challenges, the perceived lack of concern for PHC workers being ill with TB, and lack of faith in recognition reward systems appeared to have a significant impact on overall levels of motivation. This is corroborated by other studies which found that a lack of trust in the health system can act as a barrier to HCW motivation [4, 51]. In particular, a shortage of staff and poor infrastructure were singled out as health system factors with some of the greatest impacts on TB infection control implementation according to the participants.

Factors motivating compliance with TB infection control included having a colleague contract TB, fear of contagion, National Core Standard inspections, and Workmen’s Compensation. However, the power of these factors to motivate improved TB infection control practices was tempered by other concerns. For example, having a colleague fall ill with TB was regarded as a motivator but was moderated by the belief that the transmission may have in fact occurred outside of the health setting. Through the Compensation for Occupational Injuries and Diseases Act (COIDA) (Act 130 of 1993), the acquisition of TB by HCWs can be compensated should specific criteria be met [57]. However, participants indicated that they had to prove that TB infection had occurred within the workplace, which was unreasonable given that, the source of TB cannot be pinpointed and evidence in the literature [10, 58] indicates very high TB levels among HCWs. The power of the Compensation for Occupational Injuries and Diseases Act to motivate was compromised by the belief that the employer would not follow through and provide compensation.

In the context of behaviour change, as reflected in the IMB model, motivation [59] and behavioural skills [39, 60] are regarded as key. We identified numerous barriers to good infection control behaviour. Notably in our study, the implementation of specific TB infection control practices was regarded as discriminatory and stigmatising, for example separating coughing and non-coughing patients. However, the dangers of not separating TB patients from other patients, cannot be overlooked. As other studies have also found, the lack of fit -testing [60] as well as the discomfort of disposable (N95) respirators [40, 51] deter HCWs from utilising personal protective equipment controls. Poor patient compliance is a barrier to environmental control [61], while stigmatisation and discrimination are barriers to TB/HIV screening and the disclosing of one’s status [8, 21, 40, 61].

### Recommendations

The success of behaviour change models in developing strategies to improve behaviour arises from their multidimensional nature [39, 59, 60]. In this study behaviour change was seen as a prerequisite for improved TB infection control. Strategies to improve TB infection control among HCWs in PHC facilities in the Mangaung Metro derived out of our study utilising the IMB model and supported by literature are as follows:

#### Improving TB infection control knowledge

Firstly, training in TB infection control should not be limited to particular categories of staff. In accordance with guidelines from the draft National Infection Prevention and Control Strategy for TB, MDRTB and XDRTB [12] and findings from other research [51, 54] comprehensive training must be provided for all PHC staff, including support staff such as clerks and cleaners. Secondly, the knowledge gap relating to the use of disposable (N95) respirators must be targeted during training. For instance, provision of clear guidelines for the reuse and storage of respirators, providing information on the differing protection levels provided by disposable (N95) respirators versus surgical masks, etc. Thirdly, in-house training programmes should be strengthened and regular refresher training provided [51]. Fourthly, guidelines for TB infection control must be clarified [4] particularly with respect to Ideal Clinic versus National Core Standard guidelines. Finally, patient education and awareness of TB infection control measures must be addressed in order to improve compliance [21].

#### Motivating HCWs

Firstly, personal motivation can be enhanced by communicating that HCWs can be “active role-players and change agents for improved TB infection control practices” [62]. This could help overcome feelings of powerlessness which appeared to result in a laissez-faire attitude towards TB infection control - where HCWs don’t actively take ownership of infection control believing that they are completely unable to exert an influence on their environment. Secondly, social support of HCWs should be improved [29]. In particular, this could be done by highlighting the responsibility of supervisors to be positive role-models [62, 63] and by strengthening mentorship by the district health managers. The development of ‘low cost’ rewards such as certificates of compliance, to acknowledge the efforts made by individual HCWs, or alternatively rewards at facility level [39, 64] could also contribute to elevated motivation levels, particularly given that participating HCWs reported problems with formal incentive systems delivering what they promise.

#### Strengthening behavioural skills

Behavioural skills could best be improved by employing the use of practical hands-on skills training/role-playing to increase self-efficacy; for example, practice of the donning and doffing of disposable (N95) respirators [62, 63]. Lastly, efforts must be made to destigmatise TB/HIV among PHC workers and patients, as stigmatising behaviours could negate TB prevention efforts [4, 21, 39, 65].

#### Limitations of the study

As with other studies of a qualitative nature, our study was context specific and therefore findings cannot be generalised to other settings. Due to the composition of the focus group discussions, the study was limited in making a comprehensive distinction between the facility managers and TB nurses in the analysis. Finally, behavioural skills were reported and not observed. Nevertheless, our study is one of the first to draw from the voices of front-line health managers in constructing recommendations for TB infection control behaviour change in PHC settings in constrained contexts. Such participatory approaches are essential in producing contextually sensitive and relevant improvements in TB infection control behaviour change.

## Conclusions

TB infection control remains problematic in PHC facilities. HCWs need to change their behaviours and adhere to TB infection control guidelines. Behaviour change is not an easy once off event, it is a process that requires time and motivation. This study followed a participatory approach, which allowed HCWs to identify and prioritise strategies to improve TB infection control behaviours and included: training for comprehensive TB infection control for all HCWs; clarity on TB infection control policy guidelines; improved patient education and awareness of TB infection control measures; emphasis on the active role HCWs can play in infection control as change agents; improved social support; practical, hands-on training or role playing to improve behavioural skills; and the destigmatisation of TB/HIV among HCWs and patients.

## Abbreviations

HCWs: Healthcare workers
IMB: Information Motivation and Behaviour
MDR: Multi-drug resistant
PHC: Primary healthcare
TB: Tuberculosis
